# Donafenib and GSK‐J4 Synergistically Induce Ferroptosis in Liver Cancer by Upregulating HMOX1 Expression

**DOI:** 10.1002/advs.202206798

**Published:** 2023-06-17

**Authors:** Chenyang Zheng, Bo Zhang, Yunyun Li, Kejia Liu, Wei Wei, Shuhang Liang, Hongrui Guo, Kun Ma, Yao Liu, Jiabei Wang, Lianxin Liu

**Affiliations:** ^1^ Department of Hepatobiliary Surgery The First Affiliated Hospital of USTC Division of Life Sciences and Medicine University of Science and Technology of China Hefei Anhui 230001 China; ^2^ Anhui Province Key Laboratory of Hepatopancreatobiliary Surgery Hefei Anhui 230001 China; ^3^ Anhui Provincial Clinical Research Center for Hepatobiliary Diseases Hefei Anhui 230001 China; ^4^ Department of Gastrointestinal Surgery The First Affiliated Hospital of USTC Division of Life Sciences and Medicine University of Science and Technology of China Hefei Anhui 230001 China; ^5^ Hefei National Laboratory for Physical Sciences at the Microscale Division of Life Sciences and Medicine CAS Centre for Excellence in Molecular Cell Science University of Science and Technology of China Hefei Anhui 230001 China; ^6^ Department of Hepatic Surgery Key Laboratory of Hepatosplenic Surgery Ministry of Education The First Affiliated Hospital of Harbin Medical University Harbin Heilongjiang 150007 China

**Keywords:** donafenib, ferroptosis, gsk‐j4, hmox1, synthetic lethal

## Abstract

Hepatocellular carcinoma (HCC) is one of the most lethal cancers worldwide. Donafenib is a multi‐receptor tyrosine kinase inhibitor approved for the treatment of patients with advanced HCC, but its clinical effect is very limited. Here, through integrated screening of a small‐molecule inhibitor library and a druggable CRISPR library, that GSK‐J4 is synthetically lethal with donafenib in liver cancer is shown. This synergistic lethality is validated in multiple HCC models, including xenograft, orthotopically induced HCC, patient‐derived xenograft, and organoid models. Furthermore, co‐treatment with donafenib and GSK‐J4 resulted in cell death mainly via ferroptosis. Mechanistically, through integrated RNA sequencing (RNA‐seq) and assay for transposase‐accessible chromatin with high throughput sequencing (ATAC‐seq) analyses, that donafenib and GSK‐J4 synergistically promoted the expression of HMOX1 and increased the intracellular Fe2+ level is found, eventually leading to ferroptosis. Additionally, through cleavage under targets & tagmentation followed by sequencing (CUT&Tag‐seq), it is found that the enhancer regions upstream of HMOX1 promoter significantly increased under donafenib and GSK‐J4 co‐treatment. A chromosome conformation capture assay confirmed that the increased expression of HMOX1 is caused by the significantly enhanced interaction between the promoter and upstream enhancer under dual‐drug combination. Taken together, this study elucidates a new synergistic lethal interaction in liver cancer.

## Graphical abstract

A high‐throughput strategy involving a small molecule inhibitors library and a druggable CRISPR library is used to identify drugs that synergize with donafenib in the treatment of liver cancer. This study finds that donafenib and GSK‐J4 synergistically promote the expression of HMOX1 and increase the intracellular Fe^2+^ level, eventually leading to ferroptosis. This work elucidates a new synergistic lethal interaction in liver cancer.

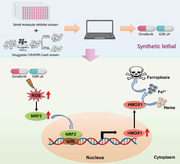

## 1 Introduction

Liver cancer is one of the most common malignant tumors worldwide and is associated with a high mortality rate.^[^ ^1^ ^]^ Over the past decades, high‐throughput sequencing approaches have indicated that the most common types of mutations in liver cancer cells, including TRET and TP53 mutations, are undruggable.^[^ ^2^ ^]^ Donafenib, a deuterated derivative of sorafenib, is an oral small molecule inhibitor of multiple receptor kinases, that is approved as the standard therapy for patients with advanced hepatocellular carcinoma (HCC). Phase II‐III clinical trial data showed that the treatment effect of donafenib was better than that of sorafenib because of its higher stability; however, there was an additional survival benefit of only 1.8 months for patients.^[^ ^3^ ^]^ Other liver cancer‐targeted drugs, including lenvatinib and regorafenib, have not achieved the clinically expected therapeutic effects.^[^ ^4^, ^5^ ^]^ These clinical studies indicate the need to find new treatments for liver cancer.

Synthetic lethality was first described by Calvin Bridges in 1922 when he observed that combinations of mutations in fruit flies led to lethality. The efficacy of drugs that target PARP in tumors with mutations in the BRCA1 and BRCA2 genes provides proof of principle that the synthetic lethality concept is clinically translatable.^[^ ^6^ ^]^ With the development of modern screening techniques, including small molecule inhibitor screening, shRNA library screening and CRISPR library screening, an increasing number of synergistic lethal combinations have been discovered tumor treatment. Regarding liver cancer, Wang et al. performed CRISPR–Cas9‐based synthetic lethality screens to determine that inhibition of MAPK1 increases the sensitivity of hepatoma cells to sorafenib^[^ ^7^ ^]^ and that pharmacological inhibition of the DNA replication kinase CDC7 induces senescence selectively in liver cancer cells with mutations in TP53.^[^ ^8^ ^]^ In addition, Jin et al. found using a kinome‐centered CRISPR–Cas9 genetic screen that EGFR inhibition is synthetic lethal with lenvatinib treatment in liver cancer.^[^ ^9^ ^]^ These results suggest that large‐scale screening is a very effective approach for identifying novel synergistic lethal interactions for tumor therapy.

Ferroptosis is a form of regulated cell death characterized by iron‐dependent accumulation of lipid hydroperoxides.^[^ ^10^, ^11^ ^]^ Dysregulation of ferroptosis is associated with various pathological conditions and diseases in humans, such as neurodegeneration, ischemia–reperfusion injury and cancer.^[^ ^12^, ^13^, ^14^, ^15^, ^16^ ^]^ Recently, substantial progress has been made in understanding the role of ferroptosis in tumor biology and cancer therapy. Multiple cancer‐related signaling pathways have been shown to control ferroptosis in cancer cells.^[^ ^17^ ^]^ Furthermore, due to their unique metabolism, high reactive oxygen species (ROS) loads, and specific mutations, some cancer cells are inherently susceptible to ferroptosis, exposing vulnerabilities that may be therapeutically targetable in certain cancer types.^[^ ^18^, ^19^, ^20^, ^21^, ^22^ ^]^ Heme oxygenase 1 (HMOX1), an inducible enzyme that oxidizes cellular heme to release biliverdin, carbon monoxide (CO) and free ferrous iron, is considered a measurable indicator of oxidative stress.^[^ ^23^ ^]^ Previous studies reported that HMOX1 performs a cytoprotective function, but accumulating evidence indicates that HMOX1 exerts cytotoxic effects when its intracellular expression level exceeds a certain threshold.^[^ ^24^, ^25^, ^26^ ^]^ Additional research is needed to verify whether ferroptosis induction by excessive activation of HMOX1 coupled with an increase in cellular labile ferrous iron could create new opportunities to effectively kill other types of tumor cells.

In this study, we sought to identify drugs that could have a synergistic effect with donafenib. Through screening of a small molecule inhibitor library and a CRISPR library, we found that GSK‐J4 has an obvious synergistic effect with donafenib. Furthermore, we found that simultaneous treatment with donafenib and GSK‐J4 can hyperactivate HMOX1 and eventually lead to ferroptosis.

## 2 Results

### 2.1 Screening of a Small Molecule Inhibitor Library and a CRISPR Library Shows that GSK‐J4 can Promote Cellular Sensitivity to Donafenib

**Figure 1:**
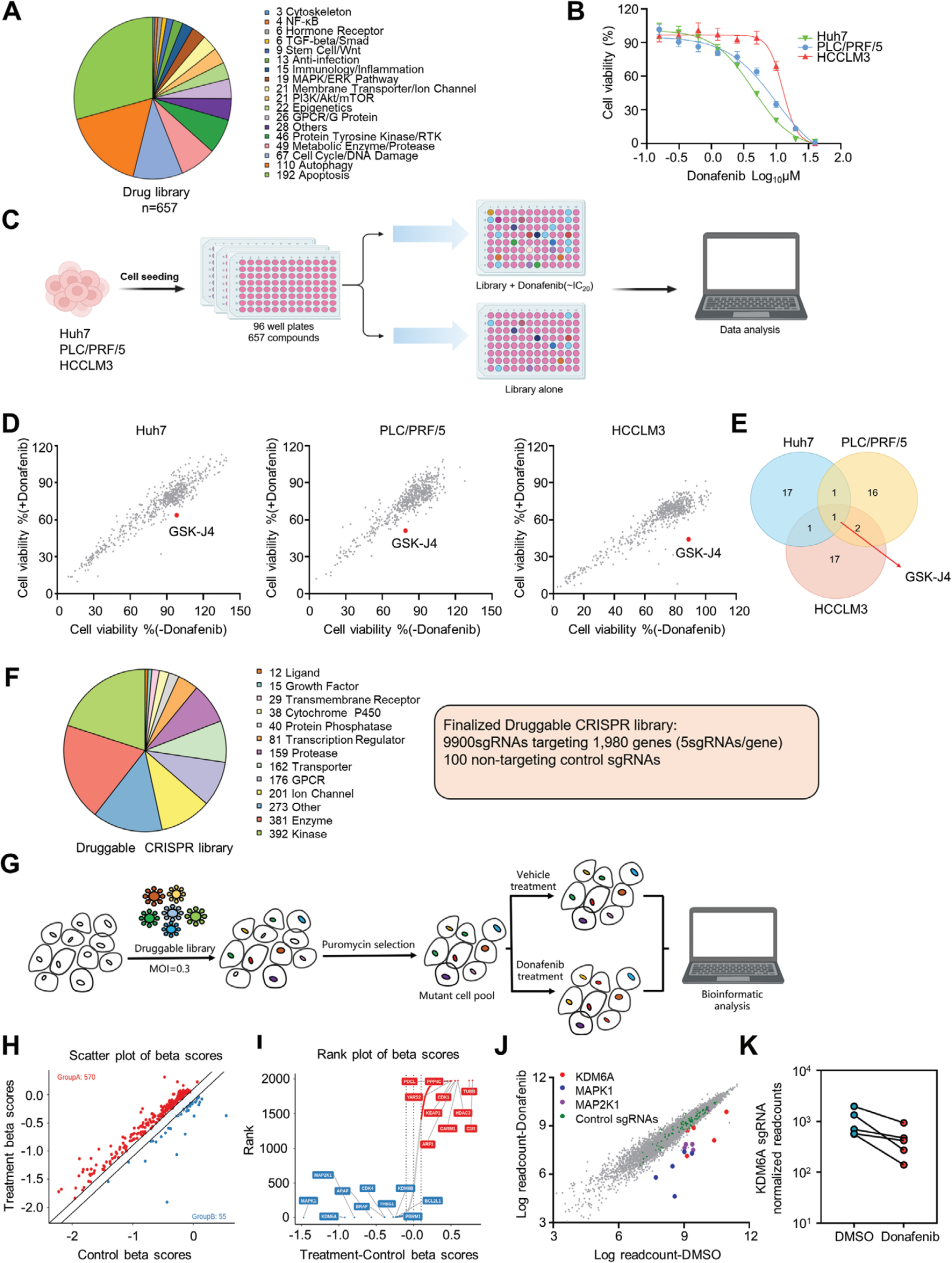
Screening of a small molecule inhibitor library and a CRISPR library shows that GSK‐J4 can promote cellular sensitivity to donafenib. A) Breakdown of the compound library based on the molecular pathways associated with the compound targets. B) Dose–response curves for three liver cancer cell lines treated with donafenib for 72 h. C) Schematic of the compound screening. D) Scatter plot showing cell viability in three liver cancer cell lines treated with compound library alone or the compound library+donafenib. The red plot shows GSK‐J4. E) Venn diagram showing the results of compound library screening in three liver cancer cell lines. F) gRNA content and distribution of protein categories in the druggable genome. G) Schematic showing the method for druggable CRISPR screening. H) Scatter plot of the treatment and control beta scores. I) Genes sorted based on the differential beta score. J) Representation of the relative abundances of the sgRNA barcode sequences. K) Readcounts of sgRNAs targeting KDM6A in the DMSO‐ and donafenib‐treated groups.

To search for drugs that could have synergistic effects on liver cancer with donafenib, we selected 657 small molecule inhibitors from the CTPR, GDSC, and PRISM databases,^[^ ^27^, ^28^, ^29^, ^30^, ^31^ ^]^ which involve various well‐known signaling pathways in cells, including the apoptosis, PI3K‐AKT, MAPK, and autophagy pathways (**Figure** **1**A and Table S1, Supporting Information). Then, based on the inhibition curves of donafenib in three liver cancer cell lines, namely, Huh7, PLC/PRF/5 and HCCLM3, we selected the approximate IC_20_ values for screening with drug library (Figure 1B,C). After analyzing the data for the three cell lines, we selected the 20 drugs with the greatest effects on each cell line for comparison. Surprisingly, only one drug – GSK‐J4 – was found to potentially have synergistic effects with donafenib in all three cell lines (Figure 1D,E). GSK‐J4 is an inhibitor of the histone demethylase KDM6A/B, which can regulate the expression of certain genes by regulating the levels of histone H3 lysine 27 di‐/trimethylation (H3K27me2/me3) in cells.^[^ ^32^ ^]^

In addition to the small molecule inhibitor library, we also designed a sgRNA library targeting components of the druggable genome. The genes in this library were based mainly on the mouse druggable CRISPR library published by the Xue laboratory,^[^ ^33^ ^]^ and some genes of interest were added through the Drug‐Gene Interaction Database (DGIdb).^[^ ^34^ ^]^ The library contains ≈10000 sgRNAs targeting 1980 human genes (5 sgRNAs per gene and 100 nontargeting control sgRNAs). Since small molecule inhibitors can be found for all proteins encoded by genes in this library, we named this library the Human Druggable CRISPR library(Figure 1F and Table S2, Supporting Information).

To systematically investigate genes whose loss increases the sensitivity of cells to donafenib, we employed pooled CRISPR–Cas9‐based negative selection screens in Huh7 cells. After transduction with the lentiviral guide RNA (gRNA) library and selection by puromycin, cells were cultured in medium containing donafenib or DMSO for 2 weeks. The gRNA sequences were polymerase chain reaction (PCR)‐amplified from the transduced cells on day 0 and after 2 weeks of culture and quantified by high‐throughput sequencing (Figure 1G). We then identified negatively selected genes by calculating the gene essentiality score using MAGeCK‐VISPR, a statistical algorithm developed for analysis of CRISPR screens.^[^ ^35^, ^36^ ^]^ Data analysis indicated that the levels of multiple sgRNAs of lethal genes decreased significantly decrease over the screening duration, demonstrating that our CRISPR library screening data are reliable (Figure S1A, Supporting Information).

The *β*‐score ranking revealed several genes that have been reported to affect the sensitivity of cells to sorafenib, including MAPK1and MAP2K1.^[^ ^7^ ^]^ In addition, we were pleasant to find that KDM6A, the target of GSK‐J4, ranked second among these genes (Figure 1H–K; Figure S1B,C, Supporting Information). By analyzing the functions and correlations of the top ten genes (in terms of synergy or resistance), we found that these genes were enriched mainly in the MAPK and chromosome regulation pathways, indicating that these two pathways are critically important for the sensitivity of cells to donafenib (Figure S1D, Supporting Information).

### 2.2 Donafenib and GSK‐J4 Exhibit Synergistic Effects in HCC Cells and Organoid

**Figure 2:**
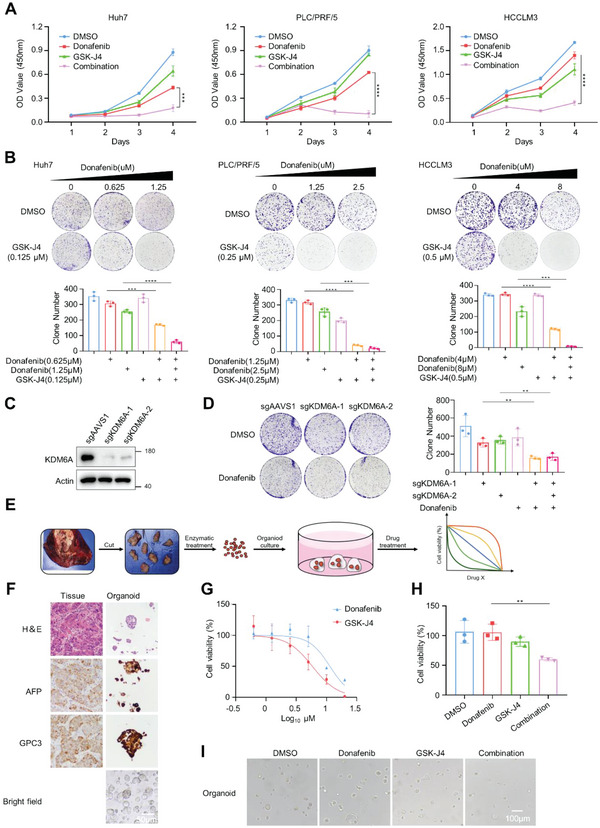
Donafenib and GSK‐J4 exhibit synergistic effects in HCC cells and organoid. A) A short‐term cell proliferation assay showed a synergistic response to donafenib in combination with GSK‐J4 in Huh7, HCCLM3 and PLC/PRF/5 cells. ***p<0.001, ****p<0.0001. B) A long‐term colony formation assay showed a synergistic response to donafenib in combination with GSK‐J4 in Huh7, HCCLM3 and PLC/PRF/5 cells. C)The level of KDM6A knockout was determined by western blotting. D) Two independent sgRNAs targeting KDM6A enhanced the response to donafenib in Huh7 cells. E) Schematic workflow of organoid generation. F) Representative images of HE and IHC staining in HCC tissues and organoids. G) Dose‐response curves for organoid treated with donafenib and GSK‐J4 for 72 h. H)The cell viability of organoid treated with DMSO, donafenib, GSK‐J4 or donafenib+GSK‐J4. **p<0.01. I) Representative micrographs of organoids treated with DMSO, donafenib, GSK‐J4 or donafenib+GSK‐J4.

To further validate our screening results, we performed validation experiments in three liver cancer cell lines. Short‐term proliferation assays and long‐term clonogenic assays confirmed the clear synergistic interaction between donafenib and GSK‐J4 (**Figure** **2**A,B). Based on this finding, we sought to determine whether these two drugs also have a similar effect in other cell lines. As expected, short‐term proliferation assays showed clear synergy between donafenib and GSK‐J4 also in HepG2, Hep3B and Hepa1‐6 cells (Figure S2A, Supporting Information). To further evaluate the synergistic effect of donafenib and GSK‐J4, we used the Bliss model to analyze the results produced by the combination of these two drugs at different concentrations, and the results showed that the synergy score between donafenib and GSK‐J4 was 18.594, which means that the interaction between two drugs is synergistic(Figure S2B, Supporting Information).^[^ ^37^ ^]^ We also confirmed that knockout of KDM6A can increase the sensitivity of Huh7 cells to donafenib in clonogenic assays, which further increased the reliability of the results indicating the synergy between donafenib and GSK‐J4 in liver cancer cell lines (Figure 2C,D).

Because of the structural similarity of donafenib with sorafenib and regorafenib, we sought to determine whether sorafenib and regorafenib also exhibit synergistic effects with GSK‐J4. Indeed, sorafenib and regorafenib exhibited synergy with GSK‐J4 in the three liver cancer cell lines (Figure S2C–E, Supporting Information).

To further determine the synergistic effect of donafenib and GSK‐J4 in preclinical models, we established patient‐derived HCC organoids for further in vitro analyses (Figure 2E). Histological analysis confirmed that these HCC organoids retained the histological features of the original tumors (Figure 2F). Consistent with our observations in liver cancer cells, donafenib and GSK‐J4 exhibited synergistic effects in patient‐derived HCC organoids. Taken together, these results show that donafenib and GSK‐J4 exhibit synergistic lethal effects in various in vitro models (Figure 2G–I).

### 2.3 Donafenib and GSK‐J4 Synergistically Suppress HCC Proliferation In Vivo

**Figure 3:**
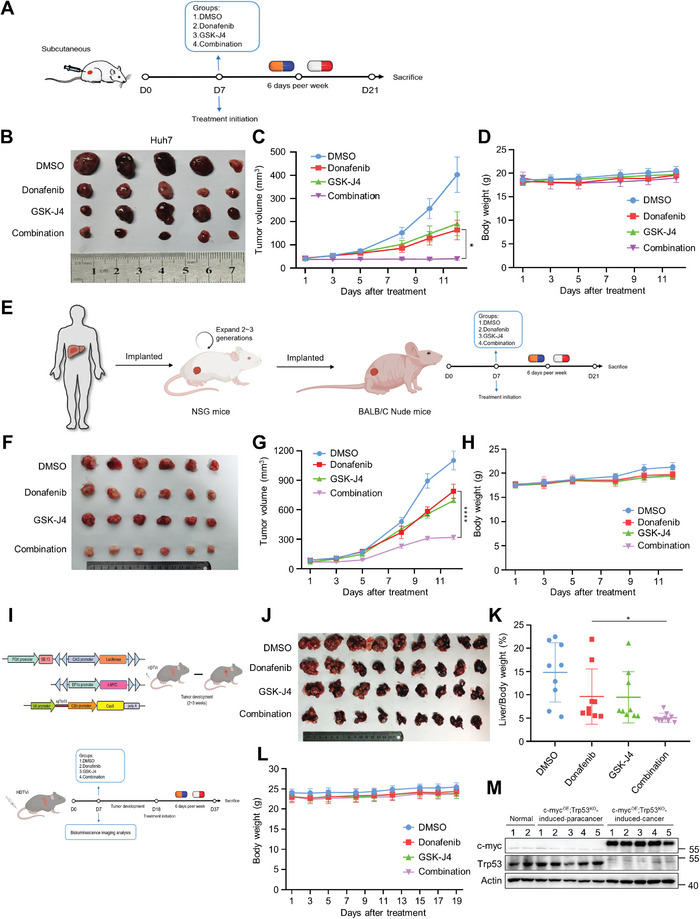
Donafenib and GSK‐J4 synergistically suppress HCC proliferation in vivo. A) Schematic representation of the in vivo anticancer effect of combined treatment with donafenib and GSK‐J4 in the subcutaneous tumor model. B) Representative images of each group of Huh7 xenografts at the end of the treatment period. C) Growth curve of each group of Huh7 xenografts. *p<0.05. D) The body weight of mice in the Huh7 xenograft model. E) Schematic representation of the in vivo anticancer effect of combined treatment with donafenib and GSK‐J4 in the PDX model. F) Representative images of tumors from each group in the PDX model at the end of the treatment period. G) Growth curve of each group in the PDX model. ****p<0.0001. H) The body weight of mice in the PDX model. I) Schematic of hydrodynamic tail vein delivery of the c‐myc proto‐oncogene transposon system and a CRISPR–Cas9 vector targeting the Trp53 tumor suppressor used to establish the model of HCC after hydrodynamic tail vein injection (HDTVi). J) Representative images of tumors from each group in the HDTVi model at the end of the treatment period. K) The liver‐to‐body ratio of each group in the HDTVi model. *p<0.05. L) The body weight of mice in the HDTVi model. M) Measurement of the expression levels of c‐myc and Trp53 in tissues of HDTVi‐induced liver cancer by western blotting.

To assess whether our in vitro findings could be reproduced in vivo, we generated xenografts from Huh7, HCCLM3, and Hepa1‐6 cells. When the tumors were palpable, the mice were randomly divided into four groups and treated with donafenib alone, GSK‐J4 alone, donafenib and GSK‐J4, or DMSO. Donafenib was administered orally, and GSK‐J4 was administered intraperitoneally. At the end of the drug treatment period, both donafenib and GSK‐J4 alone were found to slightly reduce HCC growth in vivo, while combined treatment with donafenib and GSK‐J4 completely suppressed HCC growth in vivo. Notably, the body weights of the mice in the three drug‐treated groups remained unchanged. Additionally, the mice exhibited no observable abnormal behavior during drug treatment. Collectively, these data confirm that the inhibitor GSK‐J4 can sensitize HCC cells to donafenib treatment in vivo (**Figure** **3**A–D; Figure S3A–F, Supporting Information).

Next, we used a patient‐derived xenograft (PDX) mouse model of HCC and a somatic model of HCC induced by ectopic *Myc* overexpression (OE) and homozygous *Trp53* KO (*Myc^OE^Trp53^KO^*) to verify the synergy of the two drugs in vivo. As observed in these tumor models, mice treated with donafenib or GSK‐J4 monotherapy showed a modest reduction in tumor volume, whereas combined treatment with donafenib and GSK‐J4 significantly reduced the tumor burden. Together, these results suggest that concomitant treatment with donafenib and GSK‐J4 may be a promising therapeutic approach for liver cancer (Figure 3E–M).

### 2.4 Co‐Treatment with Donafenib and GSK‐J4 Triggers Ferroptosis

**Figure 4:**
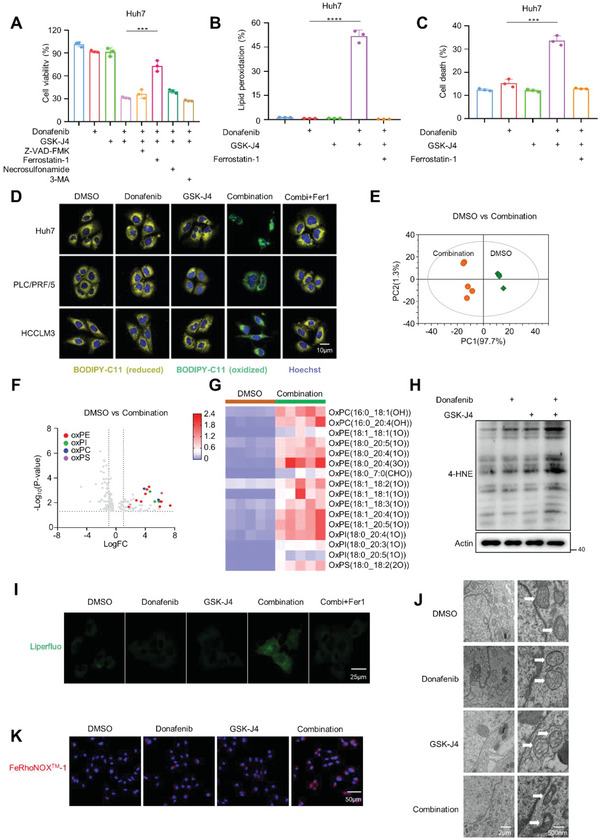
Co‐treatment with donafenib and GSK‐J4 triggers ferroptosis. A) Viability of Huh7 cells treated with donafenib, GSK‐J4 alone or in combination with Z‐VAD‐FMK, Ferrostatin‐1, Necrosulfonamide‐1 or 3‐MA. ***p<0.001. B) Lipid peroxidation measurements in Huh7 cells treated with DMSO, donafenib, GSK‐J4, donafenib+GSK‐J4 or donafenib+GSK‐J4+Fer1. ****p<0.0001. C) Cell death measurements in Huh7 cells treated with DMSO, donafenib, GSK‐J4, donafenib+GSK‐J4 or donafenib+GSK‐J4+Fer1. ***p<0.001. D) Fluorescence images of BODIPY C11‐stained Huh7, PLC/PRF/5 and HCCLM3 cells treated with DMSO, donafenib, GSK‐J4, donafenib+GSK‐J4 or donafenib+GSK‐J4+Fer1. E) Score scatter plot of OPLS‐DA model for group DMSO versus Combination in Huh7 cells. F) Volcano plot showing the upregulated peroxidized phospholipids in response to treatment with donafenib+GSK‐J4 in Huh7 cells. G) Heatmaps showing the upregulated peroxidized phospholipid species in Huh7 cells with donafenib+GSK‐J4 treatment. H) Western blotting analysis of 4‐HNE expression in Huh7 cells treated with donafenib, GSK‐J4 and donafenib+GSK‐J4. I) Fluorescence images of Liperfluo‐stained Huh7 cells treated with DMSO, donafenib, GSK‐J4, donafenib+GSK‐J4 or donafenib+GSK‐J4+Fer1.J) Transmission electron microscopy analysis of Huh7 cells treated with DMSO, donafenib, GSK‐J4 or donafenib+GSK‐J4. The white arrows indicate mitochondria. K) Fluorescence images of Huh7 cells treated with DMSO, donafenib, GSK‐J4, or donafenib+GSK‐J4.

Since significant cell death was observed during co‐treatment with donafenib and GSK‐J4, we sought to determine the type of death induced. To this end, we systematically examined the involvement of four major cell death pathways—apoptosis, ferroptosis, necrosis, and autophagy—using specific inhibitors and characteristic assays. The pancaspase inhibitor Z‐VAD‐FMK, necrostatin‐1 and 3‐methyladenine (3‐MA), which inhibit apoptosis, necrosis, and autophagic cell death, respectively, could not prevent cell death in response to donafenib+GSK‐J4 treatment. In contrast, the antioxidant ferrostatin‐1 (Fer‐1), which inhibits ferroptosis, effectively prevented cell death induced by donafenib+GSK‐J4, as assessed by different cell viability assays in multiple liver cancer cell lines (**Figure** **4**A; Figure S4A,S4D, Supporting Information). The lipid peroxidation level and the proportion of dead cells were determined by flow cytometry and confocal fluorescence microscopy. Treatment with donafenib or GSK‐J4 alone had only a slight effect, but combined treatment with the two drugs significantly increased both the intracellular lipid peroxidation level and the proportion of dead cells. The potentiating effect was reversed by cotreatment with the lipophilic antioxidant Fer‐1(Figure 4B–D; Figure S4B,C,E,F, Supporting Information). To further confirm that the co‐treatment of donafenib and GSK‐J4 can induce ferroptosis. We evaluated the levels of lipid peroxidation by performing high‐resolution liquid chromatography–mass spectrometry (LC–MS)‐based epilipidomics analysis in Huh7 and PLC/PRF/5 cells treated with DMSO or donafenib+GSK‐J4 and found that donafenib+GSK‐J4 treated cells produced a significantly increased phospholipid peroxidation(most of them are ox‐PE) compared with the cells treated with DMSO(Figure 4E–G; Figure S4G–I, Supporting Information). Further, detection of MDA levels and expression of 4‐HNE, staining of cells with Liperfluo showed that donafenib and GSK‐J4 co‐treatment could effectively increase lipid peroxidation(Figure 4H,I; Figure S4J, Supporting Information). In addition, two other ferroptosis inhibitors, DFO and Tocopherol, were also found to significantly restored cell viability in donafenib+GSK‐J4 treated cells(Figure S4K, Supporting Information). Transmission electron microscopy (TEM) revealed that Huh7 cells co‐treated with donafenib and GSK‐J4 shrunken mitochondria with an increased membrane density, which is a characteristic morphologic feature of ferroptosis (Figure 4J). And RhoNox^TM^‐1 staining showed that the intracellular Fe^2+^ level was significantly increased when cells were combined treatment with donafenib + GSK‐J4(Figure 4K; Figure S4L, Supporting Information). In conclusion, these results strongly suggest that the combination of donafenib + GSK‐J4 can induce ferroptosis in liver cancer cells.

### 2.5 Donafenib and GSK‐J4 Synergistically Induces Increased Expression of HMOX1

**Figure 5:**
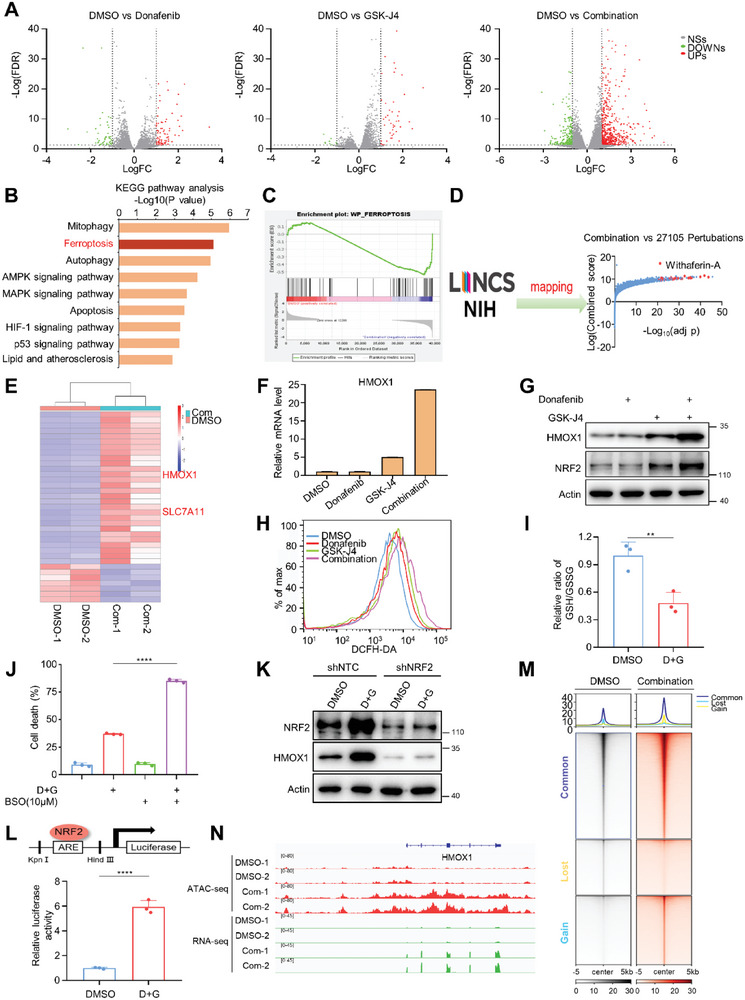
Donafenib and GSK‐J4 synergistically increased expreession of HMOX1. A) Volcano plot showing the upregulated and downregulated genes in response to treatment with donafenib, GSK‐J4 or donafenib+GSK‐J4, as determined using RNA‐seq analysis. B,C) KEGG pathway enrichment analysis and GSEA showed that genes involved in ferroptosis were significantly dysregulated under donafenib+GSK‐J4 treatment. D) Genes upregulated by donafenib+GSK‐J4 treatment were compared with gene profiles induced by treatment with chemical compounds from LINCS program. E) Heatmap of the RNA‐seq analysis results for Huh7 cells treated with DMSO or donafenib+GSK‐J4. F) qPCR analysis of HMOX1 expression in Huh7 cells treated with donafenib, GSK‐J4 or donafenib+GSK‐J4. G) Western blotting analysis of HMOX1 expression in Huh7 cells treated with donafenib, GSK‐J4 or donafenib+GSK‐J4. H) ROS level measurements in Huh7 cells treated with DMSO, donafenib, GSK‐J4 or donafenib+GSK‐J4. I) Relative ratio of GSH/GSSG in Huh7 cells treated with DMSO or donafenib+GSK‐J4. J) Cell death measurements in Huh7 cells treated with donafenib+GSK‐J4 with or without BSO(10 µm). ****p<0.0001. K) Western blotting analysis of HMOX1 and NRF2 expression in NRF2 knockdown cells treated with DMSO or donafenib+GSK‐J4. L) Relative luciferase activity of cells treated with DMSO or donafenib+GSK‐J4. M) Heatmap visualization of the normalized ATAC‐seq read coverage in Huh7 cells treated with DMSO or donafenib+GSK‐J4. N) Snapshot of the HMOX1 locus using Integrative Genomics Viewer (IGV) for ATAC‐seq and RNA‐seq data.

To further explore the mechanism underlying the synergistic efficacy of donafenib and GSK‐J4 against liver cancer, donafenib‐ and/or GSK‐J4‐treated Huh7 cells were compared by high‐throughput RNA sequencing (RNA‐seq). Co‐treatment with donafenib and GSK‐J4 strongly induced changes in intracellular expression profiles compared to those of cells treated with donafenib or GSK‐J4 alone (**Figure** **5**A). Kyoto Encyclopedia of Genes and Genomes (KEGG) pathway enrichment analysis and gene set enrichment analysis (GSEA) showed that many of the differentially expressed genes after dual‐drug treatment were enriched in the ferroptosis pathway (Figure 5B,C). To further investigate the relationship between the differential gene expression profile and ferroptosis, we integrated the genes upregulated upon drug combination with data from the Library of Integrated Network‐Based Cellular Signatures (LINCS) program. LINCS comprises tens of thousands of gene sets indicating transcriptional responses to a large library of chemical compounds.^[^ ^38^ ^]^ With this approach, we found that the genes upregulated by donafenib and GSK‐J4 co‐administration in liver cancer cells overlapped significantly with those upregulated by treatment with withaferin‐A (Figure 5D), a natural anticancer agent that can induce noncanonical ferroptosis by increasing the labile Fe^2+^ pool upon excessive activation of HMOX1.^[^ ^25^ ^]^ Reanalysis of our RNA‐seq data showed that HMOX1 expression was significantly increased in dual‐drug treated cells(Figure 5E). Both real‐time PCR and immunoblot analysis showed that HMOX1 expression was markedly increased in the dual‐drug group compared with the control and single‐drug groups(Figure 5F,G).

HMOX1 expression is controlled by the NRF2/antioxidant response element signaling pathway.^[^ ^39^ ^]^ In line with this, we observed increased levels of NRF2 in conditions in which HMOX1 is upregulated (Figure 5G). As the expression of NRF2 is mainly affected by the intracellular oxidative environment, and previous studies suggests that donafenib and GSK‐J4 can alter intracellular GSH levels, we investigated the changes in ROS and GSH levels in cells after drug treatment. The results show that co‐treatment with Donafenib and GSK‐J4 significantly increases ROS accumulation and reduces GSH level, while upregulating the expression of two genes involved in GSH synthesis (GCLC and GCLM) (Figure 5H,I; Figure S5A, Supporting Information). Moreover, we observed that co‐treatment of low concentration of BSO with donafenib+GSK‐J4 significantly increases the proportion of dead cells (Figure 5J). To demonstrate that the expression of HMOX1 is regulated by NRF2, we detected the expression of HMOX1 in shNRF2 cells treated with donafenib+GSK‐J4, and the results showed that the downregulation of NRF2 significantly blocked the upregulation of HMOX1 expression induced by the co‐treatment of donafenib and GSK‐J4 (Figure 5K). Furthermore, dual fluorescent reporter experiments revealed that NRF2 is capable of binding to the ARE sequence upstream of the HMOX1 promoter (Figure 5L).

To further confirm that NRF2 is responsible for the regulation of HMOX1 expression during co‐treatment of donafenib and GSK‐J4, we performed Assay for Transposase‐Accessible Chromatin with high‐throughput sequencing (ATAC‐seq) to map genome‐wide chromatin accessibility in cells co‐treated with donafenib and GSK‐J4.^[^ ^40^ ^]^ Combined treatment resulted in significant changes in the nucleosome‐free regions and open chromatin regions, which were located mainly in gene promoters and introns (Figure 5M; Figure S5B, Supporting Information). Regions of accessible chromatin surrounding HMOX1 were increased by drug combination, and additional peak in the upstream enhancer region was also increased. The increase in chromatin accessibility was consistent with the increased transcript level of HMOX1 (Figure 5N; Figure S5C, Supporting Information).

Transcription factor motif analysis of the ATAC‐seq data using genomic foot printing demonstrated that DNA accessibility was increased in NRF2 binding motifs (Figure S5D–F, Supporting Information). In addition, motif analysis of the upregulated genes upon dual‐drug treatment revealed that these genes were mainly enriched with NRF2 binding motifs (Figure S5G, Supporting Information). These results suggest that NRF2 is essential for the expression of HMOX1.

### 2.6 Inhibition of HMOX1 Blocks the Effects of Donafenib and GSK‐J4 on Ferroptosis

**Figure 6:**
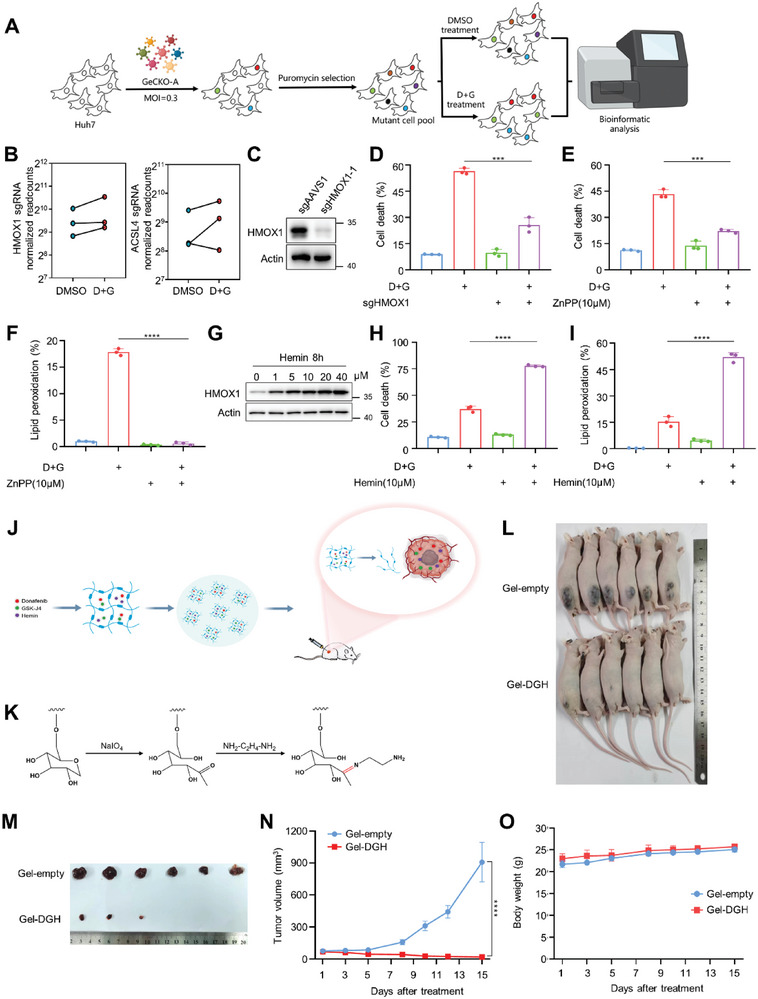
Inhibition of HMOX1 blocks the effects of donafenib and GSK‐J4 on ferroptosis. A) Schematic of genome‐wide CRISPR (GeCKO‐A) screening of Huh7 cells treated with DMSO or donafenib+GSK‐J4. B) Read counts of sgRNAs targeting HMOX1 and ACSL4 in the DMSO and donafenib+GSK‐J4 groups. C) The level of HMOX1 knockout was determined by western blotting. D) Cell death measurement in HMOX1 WT and HMOX1 knockout Huh7 cells treated with DMSO or donafenib+GSK‐J4. ***p<0.001. E) Cell death measurements in Huh7 cells treated with donafenib+GSK‐J4 with or without ZnPP. ***p<0.001. F) Lipid peroxidation measurements in Huh7 cells treated with donafenib+GSK‐J4 with or without ZnPP. ****p<0.0001. G) Western blot showing changes in HMOX1 expression in response to Hemin. H) Cell death measurements in Huh7 cells treated with donafenib+GSK‐J4 with or without Hemin. ****p<0.0001. I) Lipid peroxidation measurements in Huh7 cells treated with donafenib+GSK‐J4 with or without hemin. ****p<0.0001. J) Schematic representation of the in vivo anticancer effect of the pH‐sensitive dextran hydrogel in the subcutaneous tumor model. K) Chemical formula of the pH‐sensitive dextran hydrogel. L) Representative photographs of mice bearing tumors at the end of the treatment period. M) Representative photographs of isolated tumors at the end of the treatment period. N) Growth curve of each group in the Huh7 xenograft hydrogel‐treated model. ****p<0.0001. O) Body weight of mice in the Huh7 xenograft hydrogel‐treated model.

To confirm that HMOX1 is responsible for ferroptosis induced by co‐treatment with donafenib and GSK‐J4, we performed unbiased genome‐wide CRISPR–Cas9 screening (GeCKO‐A) in Huh7 cells treated with donafenib+GSK‐J4 or DMSO (**Figure** **6**A). We found that HMOX1 was enriched in the donafenib+GSK‐J4 group and that ACSL4, a known ferroptosis regulator involved in PUFA phospholipid synthesis, was also enriched in the donafenib+GSK‐J4 group (Figure 6B). We subsequently knocked out HMOX1 and found that the ferroptosis blockade effect was reproduced (Figure 6C,D). Similarly, pharmacological inhibition of HMOX1 activity strongly suppressed ferroptosis and lipid peroxidation after exposure to donafenib+GSK‐J4 (Figure 6E,F). In contrast, overexpression of HMOX1 resulted in cell death and increased peroxidation (Figure S6A–C, Supporting Information). Both findings indicate a cytotoxic role of HMOX1 in ferroptosis induced by donafenib and GSK‐J4 co‐treatment.

HMOX1 detoxifies heme into biliverdin, releasing carbon monoxide and Fe^2+^. Addition of HMOX1 substrate hemin induced ferroptosis more rapidly when treated in combination with donafenib+GSK‐J4 (Figure 6G–I). Based on this finding, we sought to determine the therapeutic effect of the combination of these three drugs in mice. Considering the different delivery routes of the three drugs, we used pH‐sensitive dextran hydrogels to simultaneously encapsulate these three drugs (resulting in formulation called Gel‐DGH) for in vivo experiments (Figure 6J,K). The zeta potentials and drug release rates showed that all three drugs were successfully encapsulated into the hydrogels and could respond to pH changes, indicating that in vivo experiments in mice could be performed (Figure S6D,E, Supporting Information).

Next, we verified the antitumor effect of Gel‐DGH in mice. We started treatment when the tumor volume was ≈70 mm^3^ and repeated the treatment every one or two days thereafter. By comparing the results in the two groups, we found that antitumor activity was significantly increased in Gel‐DGH‐treated mice compared with mice treated with the empty hydrogel (Figure 6L–N). No apparent adverse effects were observed, and the weights of the mice barely changed during the 15 days of observation (Figure 6O). Immunohistochemical staining of tumor sections with a proliferation marker (Ki67)‐specific antibody showed a nearly complete lack of staining in Gel‐DGH‐treated tumors compared with Gel‐empty‐treated tumors. Moreover, consistent with our proposed mechanism of action, Gel‐DGH‐treated tumors showed concomitant increases in HMOX1 and 4‐HNE staining (Figure S6F, Supporting Information).

### 2.7 Donafenib and GSK‐J4 Combination Treatment Enhances Promoter–Enhancer Interactions of HMOX1

**Figure 7:**
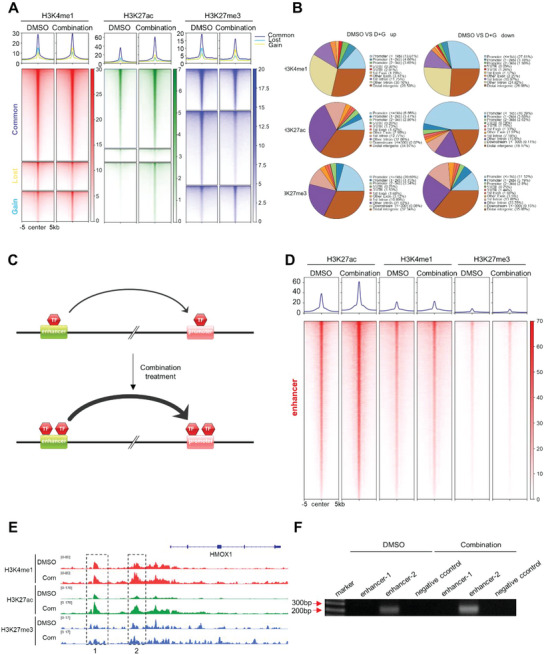
Donafenib and GSK‐J4 combination treatment enhances promoter–enhancer interactions of HMOX1. A) Heatmap indicating the CUT&Tag signal intensity of H3K4me1, H3K27ac and H3K27me3 around differentially expressed genes in Huh7 cells treated with DMSO and donafenib+GSK‐J4. B) Pie chart showing the percentages of H3K4me1, H3K27ac and H3K27me3 peaks upregulated and downregulated by donafenib+GSK‐J4 treatment in distinct genomic regions. C) Visualization of promoter–enhancer interactions. D) Heatmap showing a marked increase in overall enhancer regions in Huh7 cells treated with DMSO and donafenib + GSK‐J4. E) Snapshot of the HMOX1 locus using IGV with H3K4me1, H3K27ac and H3K27me3 CUT&Tag‐seq data. F) The 3C assay showed that the interaction of the HMOX1 promoter with upstream enhancer was enhanced upon cotreatment with donafenib and GSK‐J4.

To explain why the expression of HMOX1 increased after dual‐drug treatment, we performed Cleavage Under Targets & Tagmentation (CUT&Tag) to assess alterations in H3K27me3, H3K27ac and H3K4me1 in Huh7 cells following donafenib+GSK‐J4 co‐treatment. CUT&Tag is an enzyme tethering strategy that provides efficient high‐resolution sequencing libraries for profiling diverse chromatin components and can overcome the shortcomings of chromatin immunoprecipitation followed by sequencing (ChIP‐seq).^[^ ^41^ ^]^ As expected, we observed a global change in H3K4me1, H3K27ac and H3K27me3 in promoter and potential enhancer regions upon donafenib and GSK‐J4 combination treatment(**Figure** **7**A,B). Since changes in gene expression are closely related to enhancer modification,^[^ ^42^ ^]^ we first observed the changes in overall enhancer regions (H3K27ac+, H3K4me1+, and H3K27me3‐) after combined treatment. Surprisingly, modification of the enhancer region was significantly increased upon donafenib+GSK‐J4 co‐treatment, consistent with transcriptomic results(Figure 5A and Figure 7C,D).

We then observed changes in the above mentioned three histone modifications at the HMOX1 gene locus. As we expected, the level of H3K27me3, a transcriptionally repressive histone modification, in the promoter region of HMOX1 was significantly decreased upon dual‐drug treatment. In contrast, the levels of H3K4me1 and H3K27ac, both transcriptionally activating histone modifications, were significantly increased. In addition, surprisingly, the modification of the two putative enhancer sites upstream of the HMOX1 promoter was significantly increased after donafenib+GSK‐J4 treatment, prompting us to ask whether the upregulation of HMOX1 expression was due to enhanced interaction of the HMOX1 promoter with these enhancers upon dual‐drug treatment (Figure 7E).

We then performed chromosome conformation capture (3C) to verify the above hypothesis. Encouragingly, analysis of the results showed that the promoter of HMOX1 can indeed interact with the second upstream enhancer site and that this interaction was significantly enhanced after donafenib+GSK‐J4 treatment. This result also confirmed our hypothesis (Figure 7F).

## 3 Discussion

HCC is one of the most common malignant tumors worldwide. The development of targeted therapy for HCC has been limited because the mutations seen in HCC, such as TERT promoter mutation and TP53 mutation, are mainly untargetable. Therefore, there is an urgent need to find a new therapeutic approach for liver cancer. In this study, we found through small molecule inhibitor and druggable CRISPR library screens that donafenib and GSK‐J4 synergistically induce ferroptosis in liver cancer cells and validated this effect in various models. Furthermore, we found by integrated analysis of RNA‐seq and ATAC‐seq data that donafenib and GSK‐J4 co‐treatment significantly increased the expression of HMOX1 and explained that the increased expression of HMOX1 was due to the enhanced interaction between the HMOX1 promoter and its upstream enhancer after dual‐drug treatment.

In 2007, sorafenib became the first first‐line targeted drug approved for the treatment of inoperable liver cancer,^[^ ^43^ ^]^ and it was not until 2018 that lenvatinib was approved as the second first‐line targeted drug for the treatment of advanced liver cancer. However, lenvatinib extended patient survival by only 1.3 months.^[^ ^44^ ^]^ This finding shows that the existing treatment methods for advanced liver cancer have not achieved the desired expectations and that the discovery of new and effective treatment strategies is urgently needed. Genetic screening is a good way to find drug combinations. Regarding liver cancer, Ramona Rudalska et al. found through in vivo RNA interference (RNAi) screening that inhibition of MAPK14 downregulated intracellular RAF‐MEK‐ERK signaling to increase cell sensitivity to sorafenib, and Wang et al. screened a kinase CRISPR library and found that inhibition of MAPK1 (ERK2) can increase the sensitivity of liver cancer cells to sorafenib.^[^ ^7^, ^45^ ^]^

Ferroptosis is a unique form of cell death caused by metabolic dysfunction involving iron, lipid, oxidant and energy metabolism.^[^ ^17^ ^]^ Accumulating evidence indicates that ferroptosis contributes at least partially to the efficacy of traditional treatments, suggesting that ferroptosis can be a reliable modality for clinical treatment.^[^ ^46^, ^47^, ^48^, ^49^ ^]^ Here, we found by multiomics analysis of ferroptosis in cells that cot‐treatment with donafenib and GSK‐J4 induced the expression of HMOX1 and that either pharmacological inhibition or genetic knockdown of HMOX1 blocked the effect of donafenib+GSK‐J4 treatment, suggesting that HMOX1 is directly involved in this cell death process. Previous articles reported that HMOX1 plays a mainly protective role in cells; currently, an increasing number of articles claim that excessive HMOX1 can be toxic to cells,^[^ ^26^, ^50^ ^]^ which point wo also confirmed in our results.

In conclusion, through high‐throughput screening, we found a new synergistic lethal interaction, which provides new ideas for future liver cancer treatment.

## 4 Experimental Section

### Cell Culture

HepG2, Hep3B, HCCLM3, Huh7, PLC/PRF/5, and Hepa1‐6 cancer cells and 293T cells were obtained from the Cell Bank of the Chinese Academy of Sciences (Shanghai, China) and had been confirmed to be negative for mycoplasma contamination. All cells were cultured in a 37 °C incubator with 5% CO_2_ and maintained in culture medium supplemented with 10% Certified fetal bovine serum (Viva Cell, Shanghai, China) and 1% penicillin/streptomycin solution.

### Compounds and Antibodies

Donafenib was provided by Suzhou Zelgen Biopharmaceuticals Co, Ltd.; GSK‐J4 (HY‐15648B), sorafenib (HY‐10201A), regorafenib (HY‐10331), Z‐VAD‐FMK (HY‐16658), necrosulfonamide‐1 (HY‐100573), 3‐MA (HY‐19312), hemin (HY‐19424), BSO(HY‐106376), Tocopherol(HY‐131553), DFO(HY‐B0988) and zinc protoporphyrin (ZnPP; HY‐101193) were purchased from MCE; Fer‐1 (S7243) was purchased from Selleck; CCK‐8(C0005) was purchased from Targetmol; Liperfluo(L248), MDA Assay Kit(M496) and DCFH‐DA(R253) was purchased from Dojindo; CellTiter‐Glo® 3D Cell Viability Assay was purchased from Promega(G9681), propidium iodide (PI) was purchased from Beyotime; and FeRhoNoxTM‐1(GC901) was purchased from Goryo. Antibodies against KDM6A (ab253183), GPC3 (ab207080), NRF2 (ab62352), H3K4me1 (ab8895) and H3K27ac (ab4729) were purchased from Abcam; antibody against HA‐tag (HT301) was purchased from TransGen Biotech, antibodies against HMOX1 (10701‐1‐AP) and Actin (66009‐1‐Ig) and were purchased from Proteingroup; and antibody against H3K27me3 (07‐449) was purchased from Sigma. ATAC kit (N248) and CUT&Tag kit (N259) were purchased from novoprotein.

### Small Molecule Inhibitor Screen

A customized library consisting of 657 drugs was used for the screening. Huh7, HCCLM3, and PLC/PRF/5 cells (1000–2000 cells per well) were seeded in a 96‐well plate on day one. On day two, the cells were divided into two groups: one treated with the small molecule's library alone and the other treated with donafenib along with the small molecule's library. Cell viability was assessed 48 h after treatment. Hit compounds associated with a significant reduction in cell viability were selected for further verification. All small molecules inhibitor information was provided in Table S1 (Supporting Information).

### Design and Cloning of Druggable CRISPR sgRNA Library

The genes included in the sgRNA library were selected from papers published by Tinging Jiang et al. and DGIdb. sgRNA sequences were obtained from the Brunello whole‐genome sgRNA library or designed using the Broad Institute sgRNA Designer tool. Nontargeting control sgRNA sequences were obtained from the Brunello whole‐genome sgRNA library.^[^ ^51^ ^]^ The sgRNA library was cloned into lentiCRISPR v2 (Addgene, #52 961) using a previously described protocol.^[^ ^52^ ^]^ In brief, 5’ and 3’ flanking adapter sequences corresponding to the U6 promoter and tracrRNA sequences, respectively, were ligated to the sgRNA sequences. The oligo library was synthesized by GENEWIZ, PCR‐amplified with the Oligo‐Fwd and Oligo‐Knockout‐Rev primers using PfuUltra II Fusion HS DNA Polymerase (Agilent Technologies, #600 670), purified using a QIAquick PCR Purification Kit (QIAGEN, #28 104), and finally inserted into the BsmBI‐digested lentiCRISPR v2 vector using Gibson assembly. To determine the sgRNA distribution in the cloned library, the sgRNA target region was amplified with the NGS‐Lib‐Fwd and NGS‐Lib‐KO‐Rev primers, size‐selected on a 2% agarose gel, purified using a QIAquick Gel Extraction Kit (QIAGEN, #28 704), and then submitted for sequencing on the Illumina HiSeq 2500 system (100‐nt single‐end reads) at GENEWIZ. All genes and sgRNAs information was provided in Table S2 (Supporting Information). The CRISPR library constructed in this study had been submitted to addgene.

### Pooled Synthetic Lethal CRISPR Screen

Huh7 cells were infected at a multiplicity of infection of 0.3 and selected with puromycin. An initial sample of 5 million (500X) cells was harvested, and infected cells were cultured for ≈14 population doublings under each condition (treatment with DMSO or 3 µm donafenib). Final samples of 5 million cells were collected. DNA was extracted (*Quick*‐DNA™ Midiprep Plus Kit, ZYMO, D4075). The sgRNA inserts were amplified and barcoded by PCR using unique primers for each condition. PCR amplicons were purified and sequenced (Illumina HiSeq 2500). Enrichment of sgRNAs and statistical analyses were performed with MAGeCK (v0.5.6).

### Construction of sgRNA Plasmids

For construction of sgRNA expression plasmids, DNA oligonucleotides were annealed and ligated into the BsmBI‐digested LentiCRISPRv2 vector. The sgRNA targeting sequences were listed in Table S3 (Supporting Information).

### Cell Proliferation Assay

The cell proliferation assay was performed using a CCK‐8 Kit (Dojindo). A total of 1000–2000 cells were seeded in each well of a 96‐well plate. CCK‐8 solution (10 µL) was added to 100 µL of culture medium, and the optical density was measured at 450 nm. The obtained values were normalized to that of the blank well. Then, relative cell viability was determined by normalization to the corresponding DMSO‐treated wells. GraphPad Prism 8 software was used to plot the fitted regression curves.

### Long‐Term Clonogenic Assay

Cells were cultured and seeded in 6‐well plates at a density of 1000–3000 cells per well, depending on the growth rate, and were cultured in medium containing the indicated drugs for 10–14 days (the medium was changed twice weekly). The cells were fixed with 4% formaldehyde in PBS and stained with 0.1% crystal violet diluted in water.

### Western Blotting

RIPA buffer supplemented with protease inhibitor cocktail was used to lyse cells on ice for 1 h, and the protein concentration was determined using a BCA assay kit. Extracted proteins were denatured by boiling at 95 °C for 10 min and were then subjected to electrophoresis on 8%–12% SDS–polyacrylamide gels.

### Organoid Culture

Organoids were generated according to previously described protocols with slight modifications.^[^ ^53^ ^]^ Briefly, one‐quarter of the patient‐derived cancer tissue was minced into small pieces of ≈1 mm^3^ and incubated at 37 °C with digestion solution. Incubation was performed for 30 min to 3 h. Digestion was stopped when no pieces of tissue were left, and the suspension was then filtered through a 100‐µm nylon cell strainer and centrifuged for 5 min at 200 × g. Digested cells were subsequently cultured in liver cancer organoid medium (M103, Accurate International Biotechnology Co., Ltd.). To use the clinical materials for research purposes, both the patients previously obtained written informed consent and the research approval of the Institutional Research Ethics Committee of the First Affiliated Hospital of University of Science and Technology of China were obtained. Patient volunteered and received no compensation. Patient information was provided in Table S4 (Supporting Information) (Permit Number: 2022KY‐178).

### Animal Experiments

All animal experiments were undertaken and conducted with approval from the Animal Research Ethics Committee of the University of Science and Technology of China. (Permit Number: USTCACUC22220122021).

For the xenograft experiment, 3 × 10^6^ cells were subcutaneously injected into 5‐week‐old male BALB/c nude mice followed with the indicated treatment. Mice were randomized into different groups at the time of treatment initiation. The tumor size was measured by caliper every two days. The tumor volume was calculated as follows: (length×width^2^)×0.5.

For the PDX experiment, samples from liver cancer patients were cut into small pieces, transplanted into severely immunodeficient(NOG) mice, and then passed into NOG and nude mice for 3 generations separately. Finally, the tumors were transplanted into nude mice for experiments. Mice were randomized into different groups at the time of treatment initiation. The tumor size was measured by caliper every two days. The tumor volume was calculated as follows: (length×width^2^)×0.5. The patients previously obtained written informed consent and the research approval of the Institutional Research Ethics Committee of the First Affiliated Hospital of University of Science and Technology of China were obtained. Patient volunteered and received no compensation. Patients' information was provided in Table S4 (Supporting Information).

For the orthotopic liver cancers model, A sterile 0.9% NaCl solution/plasmid mixture containing 20 µg of the pT3‐EF1a‐Myc plasmid, 20 µg of px330‐p53 and 5 µg of PT2/C‐Luc//PGK‐SB13 was delivered to 8‐week‐old C57BL/6 mice by hydrodynamic tail vein injection. Approximately three weeks after induction, the mice were randomly divided into four groups. All mice were euthanized after 20 days of drug treatment.

### Analysis of Cell Death by PI Staining

A total of 1×10^5^ cells per well were seeded in six‐well plates one day prior to treatment with drugs. On the next day, cells were treated to induce ferroptosis; the treated cells were then harvested by trypsinization and resuspended in fresh PBS containing PI. After incubation for 10 min, the cells were analyzed by flow cytometry. Data were analyzed using FlowJo software.

### Analysis of Lipid Peroxidation by Flow Cytometry and Imaging

For flow cytometric analysis, cells were harvested and washed with PBS, resuspended in PBS containing 5 µm BODIPY 581/591 C11 dye and incubated in a tissue culture incubator for 30 min. The cells were then washed twice with PBS and resuspended in 200 µL of PBS. ROS levels were analyzed using a BD FACS instrument in the FL1 channel, and data were analyzed using FlowJo. In each sample, 5000 cells were analyzed.

For imaging, cells (1×10^4^ cells per dish) were plated in 3.5 mm dishes and incubated for the indicated times. After treatment, the cells were incubated in medium containing 1 µg mL^−1^ Hoechst 33 342 (Beyotime) and 5 µm BODIPY 581/591 C11 for live‐cell imaging. The cells were imaged at 40× magnification using a Leica confocal microscope. All images were acquired with the same instrument parameters and processed with the same settings to maximize the ability to compare results between conditions.

### Epilipidomics Analysis

Lipids were extracted from cells using the methyl‐tert‐butyl ether (MTBE) method. In brief, cells were extracted in ice‐cold methanol (200 µL) containing dibutylhydroxytoluene (BHT, 5 µg mL^−1^) and SIL‐IS (20 µL) by ultrasonication for 10 min in ice‐water bath. The cell lysates were added by 1 mL MTBE, vortexed 30 s, and kept at 4 °C for 10 min. Phase separation was performed by an addition of 200 µL water and centrifugation at 5000 g and 4 °C for 10 min. The organic phase was collected and dried under gentle nitrogen gas. The dried residues were redissolved in 100 µL of dichloromethane/methanol (1:1, v/v) and transferred to glass insert vial for UHPLC‐HRMS/MS analysis with injection of 5 µL.

Chromatographic separation was performed on a ThermoFisher Ultimate 3000 UHPLC system with a Waters CSH C18 column (2.1 mm × 100 mm, 1.7 µm) at 30 °C. The mobile phases consisted of (A) water and (B) isopropanol/acetonitrile (9:1, v/v), both with 10 mm ammonium format and 0.1% formic acid. A linear gradient elution was performed with the following program: 0 – 1 min, 40% B; 4 min, 65% B; 10 min, 75% B; 15 min, 95% B and held to 17 min; 11.1 min, 40% B and held to 20 min. The flow rate was 0.25 mL min^−1^, and the injection volume was 2 µL both for positive mode and negative mode.

Mass spectrometry analysis was performed on ThermoFisher Q Exactive Hybrid Quadrupole‐Orbitrap Mass Spectrometry in Heated Electrospray Ionization Negative (HESI‐) mode with the following settings: spray voltage 3.5 kV, capillary temperature 350 °C, and aux gas heater temperature 150 °C, sheath gas 33 arbitrary units, aux gas 10 arbitrary units, S‐Lens RF level 50%. The full scan was operated at a high‐resolution of 70 000 FWHM (m/z = 200) at a range of 130 – 1950 m/z with AGC target setting at 1×106. Simultaneously, the fragment ions information of top 10 precursors each scan was acquired by Data‐dependent acquisition (DDA) with HCD stepped normalized collision energy (stepped NCE) at 20, 30 and 40%, mass resolution of 17 500 FWHM, and AGC target of 1×10^5^.

The raw data of UHPLC‐HRMS/MS were preprocessed by Compound Discoverer (version 3.3, Thermo Fisher) with lipidomic workflow template, where retention times alignment, compound detection, and compound group were performed. In addition to the default parameters, other main settings were as follows, Mass tolerance 5 ppm, RT tolerance 0.2 min. The final data was exported to a peak table file including sample names, accurate mass to charge (m/z) of precursors, retention times, and peak areas. Subsequently, lipid identification was performed by LipidMatch 3.0 with default parameters and in‐silico lipid library with format as adduct. All lipids including oxidized lipids with level 1 matching including precursor ion (MS1) and fragment ions (MS/MS) were identified as final lipids. The peak area data was normalized against the peak areas of SIL‐ISs, and analyzed in R platform, where parametric test was performed on the data of normal distribution by Welch's t test, while nonparametric test was performed on the data of abnormal distribution by Wilcoxon Mann‐Whitney test. The *p* values of univariate statistical analysis lower than 0.05 and the absolute value of binary logarithm of fold change (LFC) larger than 0.263 (1.2 fold) were identified as potential differential lipids.

### Detection of MDA Assay

The relative MDA concentration in cells was assessed using MDA Assay Kit (Dojindo) according to the manufacturer's instructions. Briefly, MDA in the sample reacts with thiobarbituric acid (TBA) to generate an MDA‐TBA adduct. The MDA‐TBA adduct can be quantified according to the fluorescence intensity (Ex/Em = 540/590).

### Detection of Labile Iron by Imaging and Flow Cytometry

For flow cytometric analysis, cells were harvested and washed with HBSS, resuspended in HBSS containing 5 µm FeRhoNox^TM^‐1 and incubated in a tissue culture incubator for 30 min. The cells were then washed twice with HBSS and resuspended in 200 µL of HBSS. Labile iron levels were measured using a BD FACS instrument, and data were analyzed using FlowJo. In each sample, 5000 cells were analyzed.

For imaging, cells (1×10^4^ cells per dish) were plated in 3.5 mm dishes and incubated for the indicated times. After treatment, the cells were incubated with medium containing 1 µg mL^−1^ Hoechst 33 342 (Beyotime) and 5 µm FeRhoNox^TM^‐1 for live‐cell imaging. Cells were imaged using a Leica confocal microscope. All images were acquired with the same instrument parameters and processed with the same settings to maximize the ability to compare results between conditions.

### Analysis of Lipid Peroxidation Using Liperfluo

Cells (1×10^4^ cells per dish) were plated in 3.5 mm dishes and incubated for the indicated times. After treatment, the cells were incubated in medium containing 10 µm Liperfluo (Dojindo) for live‐cell imaging. The cells were imaged at 40× magnification using a Leica confocal microscope. All images were acquired with the same instrument parameters and processed with the same settings to maximize the ability to compare results between conditions.

### TEM

Huh7 cells were fixed first with 2.5% glutaraldehyde for 12 h at 4 °C and then with 2% osmium tetroxide. After washing, the samples were stained with 1% uranyl acetate aqueous solution; dehydrated sequentially in 50, 70, 90, 95, and 100% ethanol; and immersed in Eponate 12 resin. The samples were then cut into ultrathin sections and counterstained with uranyl acetate and lead citrate. Finally, images were acquired using a transmission electron microscope (120 kV; Tecnai G2 Spirit, FEI).

### RNA‐Seq and Data Analysis

Total RNA was extracted from cells by using an RNAprep Pure Micro Kit from Tiangen. Libraries were generated using the NEB Next Ultra RNA Library Prep Kit for Illumina. RNA‐seq was performed on Illumina NovaSeq platform by Novogene. Reads were first aligned to the human reference genome hg19, transcripts were assembled with HTSeq (v0.6.1), and gene expression analysis was performed using DESeq2. Pathway enrichment analysis was performed with Enricher.^[^ ^54^ ^]^

### qPCR

According to the manufacturer's instructions, total RNA was extracted from cells by using an RNAprep Pure Micro Kit from Tiangen and complementary DNA was synthesized from 500 ng of RNA using PrimeScript™ RT Master Mix (TAKARA). qPCR was performed using SYBR Green Master Mix (TAKARA). All samples were normalized to housekeeping genes Actin. Primer sequences are shown in Table S5 (Supporting Information).

### Detection of ROS Assay

A total of 1×10^5^ cells per well were seeded in six‐well plates one day prior to treatment with drugs. On the next day, cells were treated to induce ferroptosis; the treated cells were then harvested by trypsinization and resuspended in loading buffer containing DCFH‐DA(Dojindo) and incubated in a tissue culture incubator for 30 min. The cells were then washed twice with HBSS and resuspended in 200 µL of HBSS. ROS levels were analyzed using a BD FACS instrument in the FL1 channel, and data were analyzed using FlowJo.

### Detection of GSH

To detect the GSH level, 1 mL of 80% methanol (including 10 mm ammonium bicarbonate and 50 mm NEM) was added to cells. Following processing by 5 cycles of 1 min ultra‐sonication and 1 min interval in ice‐water bath, the extraction solution was centrifuged at 15 000 *g* and 4 °C for 15 min. The 200 µL supernatant was evaporated to dryness under nitrogen gas and reconstituted in 50 µL of 50% acetonitrile (including 1 µg mL^−1^ pnenylalanine‐d_5_) prior to perform UHPLC‐HRMS/MS analysis. Quality control (QC) sample was obtained by isometrically pooling all the prepared samples.

All the analytical standards and internal standards were prepared individually at the concentration of 1 mg mL^−1^ as stock solution. A standard working solution of 20 µg mL^−1^ for each standard in 50% acetonitrile was prepared by mixing each standard stock solution (1 mg mL^−1^). The standard working solution was then serially diluted to cover a range from 20–20 000 ng mL^−1^. The samples of calibration curves were finally prepared by isometrically mixing the serially diluted standard solution with internal standards solution to generate calibration levels covering a range of 10–10 000 ng mL^−1^.

The UHPLC‐MS/MS analysis was performed on an Agilent 1290 Infinity II UHPLC system coupled to a 6470A Triple Quadrupole mass spectrometry (Santa Clara, CA, United States). Samples was injected onto a Waters ACQUITY UPLC® BEH Amide column (100 mm × 2.1 mm, 1.7 µm) at a flow rate of 0.25 mL min^−1^, and the injection volume was 1 µL. The mobile phase consisted of 95% acetonitrile (phase A) and aqueous ammonium acetate (15 mm) at pH9 (phase B). The chromatographic separation was conducted by a gradient elution program as follows: 1 min, 5% A; 14 min, 30% A; 15 min, 50% A; 17 min, 50% A; 17.5 min, 5% A; 20 min, 5% A.

The eluted analysts were ionized in an electro spray ionization source in positive mode (ESI^+^). The temperature of ESI^+^ source drying gas was 300 °C and sheath gas was 350 °C. The flow rate of ESI^+^ source drying gas and sheath gas were 5 and 11 L min^−1^, respectively. The pressure of nebulizer was 45 psi, and capillary voltage was 4000 V. The dynamic multiple reaction monitoring (dMRM) was used to acquire data in optimized MRM transition (precursor → product). The total scan time per cycle was 300 ms. The Agilent MassHunter software (version B.08.00) was used to control instruments and acquire data.

The raw data was processed by Agilent MassHunter Workstation Software (version B.08.00) by using the default parameters and assisting manual inspection to ensure the qualitative and quantitative accuracies of each compound. The peak areas of target compounds were integrated and output for quantitative calculation.

### Luciferase Reporter Assay

Cells were seeded into 24‐well plates. After overnight incubation, HEK293T cells were transfected with 4 ng of pSV‐Renilla plasmid and 100 ng of firefly luciferase reporter plasmid. After 48 h, the cells were lysed and tested using a dual‐luciferase reporter assay kit (Promega) according to the manufacturer's instructions. Independent experiments were conducted in triplicate.

### Assay for Transposase‐Accessible Chromatin with high throughput sequencing(ATAC‐Seq) and Data Analysis

Library preparation for ATAC‐seq was based on a previous protocol with slight modifications.^[^ ^55^ ^]^ In brief, freshly cultured Huh7 cells (100 000 per sample) were harvested and washed with 500 µL of cold PBS containing a protease inhibitor. Nuclei were collected by centrifugation at 500 × g for 5 min at 4 °C after the cell pellets were resuspended in lysis buffer (10 mm Tris‐Cl (pH 7.4), 10 mm NaCl, and 3 mm MgCl_2_ containing 0.1% NP‐40, 0.1% Tween 20 and 0.01% digitonin). Nuclei were incubated with Tn5 transposase in transposase reaction mix buffer (Illumina) for 1 h at 37 °C. DNA was purified using magnetic beads and quantified with a Qubit dsDNA HS Assay Kit. PCR amplification was performed with High‐Fidelity 5× PCR Master Mix [72 °C/3 min + 98 °C/30 s + 10 × (98 °C/15 s + 60 °C/15 s + 72 °C/8 s) + 72 °C/2 min]. The libraries were purified using magnetic beads. The ATAC‐seq libraries were then subjected to paired‐end sequencing on the HiSeq 2500 platform (Illumina). Trimmed reads were first aligned to the human reference genome hg19 using Bowtie2, peak calling on nucleosome‐free reads was enhanced with MACS2, and the differential peaks between the samples were analyzed with edgeR.

### Genome‐Wide CRISPR‐Cas9 Screen

Huh7 cells were transduced with a human CRISPR KO lentiviral library (GeCKO‐A)^[^ ^56^ ^]^ at a multiplicity of infection of 0.3 and selected with puromycin. An initial sample of 12 million cells (200×) was harvested, and the remaining transduced cells were divided into two groups. One group was subjected to three cycles of treatment with donafenib+GSK‐J4 for 24 h and replacement of the drug‐containing medium with DMEM every 2 days, and the other group was left untreated as the control. Final samples containing 12 million cells were collected. DNA was extracted (*Quick*‐DNA™ Midiprep Plus Kit, ZYMO, D4075). The sgRNA inserts were amplified and barcoded by PCR using unique primers for each condition. The PCR amplicons were purified and sequenced (Illumina HiSeq 2500 system). Enrichment of sgRNAs and statistical analyses were performed with MAGeCK (v0.5.6). Human GeCKO v2 library was a gift from Feng Zhang.^[^ ^57^ ^]^

### Synthesis of Gel‐DGH

Dextran (DEX; 5 g) was dissolved in 30 mL of pure water, put on a magnetic stirrer, and stirred until clear Then, NalO4 (2.5 g) was added, and the reaction was allowed to proceed at 50 °C for 4 h. At the end of the reaction time, 5 g of glycerol was added to stop the reaction, and the mixture was placed into a dialysis bag with a molecular weight of 8,000 kD, dialyzed for 36 h, and then poured into a beaker. Then, 3 mL of ethylenediamine was added, stirred at room temperature (RT) for 12 h, placed into a dialysis bag with a molecular weight of 8,000 kD, and dialyzed for 36 h. Finally, the samples were freeze‐dried to prepare pH‐sensitive hydrogels. The freeze‐dried hydrogels were dissolved in water to an appropriate concentration, and donafenib and hemin were dissolved in DMSO to the required concentration and stirred at RT for 2 h. Then, donafenib and hemin were put into an 8,000 kD dialysis bag for dialysis for 36 h in pure water and freeze‐dried for later use. After GSK‐J4 was dissolved to the required concentration, the dried hydrogel was added and stirred at RT for 2 h to yield the hydrogel containing the required concentration of the three drugs.

### Hematoxylin & Eosin (H&E) and Immunohistochemist (IHC) Staining

Samples were dewaxed with xylene and then rehydrated through a graded ethanol series. After antigen retrieval, the sections were incubated with 3% hydrogen peroxide for 1 h to block endogenous peroxidase activity. Next, the sections were preincubated in 3% BSA for 1 h to prevent nonspecific staining, and were then incubated with anti‐Ki67, anti‐HMOX1 or anti‐4‐HNE antibodies at 4 °C overnight. The next day, the sections were incubated with a secondary antibody followed by diluted DAB Chromogen solution.

### Cleavage Under Targets & Tagmentation followed by sequencing (CUT & Tag‐Seq )and Data Analysis

Library preparation for CUT & Tag‐seq was based on a previous protocol with slight modifications.^[^ ^41^ ^]^ Approximately 100 000 Huh7 cells were harvested and washed with wash buffer (20 mm HEPES (pH 7.5), 150 mm NaCl, 0.5 mm spermidine, and 1× protease inhibitor cocktail). After centrifugation, the cells were resuspended in 90 µL of buffer, and 10 µL of ConA beads were added at RT for 10 min. Bead‐bound cells were collected by placing a tube on a magnetic stand and removing the clear liquid. The bead‐bound cells were resuspended in 50 µL of primary antibody buffer (20 mm HEPES (pH 7.5), 150 mm NaCl, 0.5 mm spermidine, 1× protease inhibitor cocktail, 0.05% digitonin, and 2 mm EDTA) and incubated with antibodies at RT. Unbound primary antibodies were removed by placing the tube on the magnetic stand and removing the liquid. The primary antibody/bead‐bound cells were mixed with 100 µL of antibody buffer containing anti‐rabbit IgG (diluted 1:100) for 1 h at RT. Bead‐bound cells were washed using 1 mL of antibody buffer to remove unbound antibodies. A 1:100 dilution of the pA‐Tn5 adapter complex was prepared in ChiTag buffer (20 mm HEPES (pH 7.5), 300 mm NaCl, 0.5 mm spermidine, 0.05% digitonin, and 1× protease inhibitor cocktail). After removing the liquid from the samples on the magnetic stand, 100 µL of a mixture of pA‐Tn5 was added to the bead‐bound cells, vortexed gently, and incubated at RT for 1 h. After the addition of 1 mL of ChiTag buffer to remove unbound pA‐Tn5 protein, the cells were resuspended in 50 µL of tagmentation buffer (10 mm MgCl2 in ChiTag buffer) and incubated at 37 °C for 1 h. One microliter of 10% SDS was added to stop tagmentation and incubated at 55 °C for 1 h. DNA was then extracted using magnetic beads. Sequencing libraries were prepared using NEBNext HiFi 5× PCR Master Mix according to the manufacturer's instructions. The PCR products were cleaned up with DNA Clean Beads and quantified using a Qubit dsDNA HS Assay Kit (Agilent Technologies). The libraries were sequenced on the HiSeq 2500 platform (Illumina). Trimmed reads were first aligned to the human reference genome hg38 using Bowtie2, narrow peaks were called by MACS2, and differential peaks between samples were analyzed with edgeR.

### Chromosome Conformation Capture (3C)

A 3C assay was used to detect interactions between promoters and enhancers. The 3C assay was performed as described previously.^[^ ^58^ ^]^ BfaI was used for genomic DNA digestion. The primers used in this assay were listed in Table S6 (Supporting Information).

## Conflict of Interest

The authors declare no conflict of interest.

## Author Contributions

C.Z., B.Z. and Y.L. contributed equally to this work. C.Z. was responsible for data acquisition, analysis and drafting the article; B.Z. and Y.L. performed the in vivo experiments. K.L., W.W., S.L., H.G. and K.M. provided advice for the design of the study. L.L., J.W. and L.L. were responsible for the conception, design and supervision of the study.

## Supporting information

Supporting Information

## Data Availability

The data that support the findings of this study are available from the corresponding author upon reasonable request.

## References

[1] J. M. Llovet , R. K. Kelley , A. Villanueva , A. G. Singal , E. Pikarsky , S. Roayaie , R. Lencioni , K. Koike , J. Zucman‐Rossi , R. S. Finn , Nat. Rev. Dis. Primers 2021, 7, 6. 33479224 10.1038/s41572-020-00240-3 PMC13537433

[2] J. Zucman‐Rossi , A. Villanueva , J. C. Nault , J. M. Llovet , Gastroenterology 2015, 149, 1226. 26099527 10.1053/j.gastro.2015.05.061

[3] S. Qin , F. Bi , S. Gu , Y. Bai , Z. Chen , Z. Wang , J. Ying , Y. Lu , Z. Meng , H. Pan , P. Yang , H. Zhang , X. Chen , A. Xu , C. Cui , B. Zhu , J. Wu , X. Xin , J. Wang , J. Shan , J. Chen , Z. Zheng , L. Xu , X. Wen , Z. You , Z. Ren , X. Liu , M. Qiu , L. Wu , F. Chen , J. Clin. Oncol. 2021, 39, 3002. 34185551 10.1200/JCO.21.00163 PMC8445562

[4] J. Bruix , S. Qin , P. Merle , A. Granito , Y. H. Huang , G. Bodoky , M. Pracht , O. Yokosuka , O. Rosmorduc , V. Breder , R. Gerolami , G. Masi , P. J. Ross , T. Song , J. P. Bronowicki , I. Ollivier‐Hourmand , M. Kudo , A. L. Cheng , J. M. Llovet , R. S. Finn , M. A. LeBerre , A. Baumhauer , G. Meinhardt , G. Han , R. Investigators , Lancet 2017, 389, 56. 27932229 10.1016/S0140-6736(16)32453-9

[5] K. Ikeda , M. Kudo , S. Kawazoe , Y. Osaki , M. Ikeda , T. Okusaka , T. Tamai , T. Suzuki , T. Hisai , S. Hayato , K. Okita , H. Kumada , J. Gastroenterol. 2017, 52, 512. 27704266 10.1007/s00535-016-1263-4 PMC5357473

[6] P. C. Fong , D. S. Boss , T. A. Yap , A. Tutt , P. Wu , M. Mergui‐Roelvink , P. Mortimer , H. Swaisland , A. Lau , M. J. O'Connor , A. Ashworth , J. Carmichael , S. B. Kaye , J. H. Schellens , J. S. de Bono , N. Engl. J. Med. 2009, 361, 123. 19553641 10.1056/NEJMoa0900212

[7] C. Wang , H. Jin , D. Gao , C. Lieftink , B. Evers , G. Jin , Z. Xue , L. Wang , R. L. Beijersbergen , W. Qin , R. Bernards , J. Hepatol. 2018, 69, 1057. 30030148 10.1016/j.jhep.2018.07.004

[8] C. Wang , S. Vegna , H. Jin , B. Benedict , C. Lieftink , C. Ramirez , R. L. de Oliveira , B. Morris , J. Gadiot , W. Wang , A. du Chatinier , L. Wang , D. Gao , B. Evers , G. Jin , Z. Xue , A. Schepers , F. Jochems , A. M. Sanchez , S. Mainardi , H. Te Riele , R. L. Beijersbergen , W. Qin , L. Akkari , R. Bernards , Nature 2019, 574, 268. 31578521 10.1038/s41586-019-1607-3 PMC6858884

[9] H. Jin , Y. Shi , Y. Lv , S. Yuan , C. F. A. Ramirez , C. Lieftink , L. Wang , S. Wang , C. Wang , M. H. Dias , F. Jochems , Y. Yang , A. Bosma , E. M. Hijmans , M. H. P. de Groot , S. Vegna , D. Cui , Y. Zhou , J. Ling , H. Wang , Y. Guo , X. Zheng , N. Isima , H. Wu , C. Sun , R. L. Beijersbergen , L. Akkari , W. Zhou , B. Zhai , W. Qin , et al., Nature 2021, 595, 730. 34290403 10.1038/s41586-021-03741-7

[10] S. J. Dixon , K. M. Lemberg , M. R. Lamprecht , R. Skouta , E. M. Zaitsev , C. E. Gleason , D. N. Patel , A. J. Bauer , A. M. Cantley , W. S. Yang , B. Morrison , 3rd, B. R. S. , Cell 2012, 149, 1060. 22632970 10.1016/j.cell.2012.03.042 PMC3367386

[11] B. R. Stockwell , J. P. Friedmann Angeli , H. Bayir , A. I. Bush , M. Conrad , S. J. Dixon , S. Fulda , S. Gascon , S. K. Hatzios , V. E. Kagan , K. Noel , X. Jiang , A. Linkermann , M. E. Murphy , M. Overholtzer , A. Oyagi , G. C. Pagnussat , J. Park , Q. Ran , C. S. Rosenfeld , K. Salnikow , D. Tang , F. M. Torti , S. V. Torti , S. Toyokuni , K. A. Woerpel , D. D. Zhang , Cell 2017, 171, 273. 28985560 10.1016/j.cell.2017.09.021 PMC5685180

[12] L. Chen , W. S. Hambright , R. Na , Q. Ran , J. Biol. Chem. 2015, 290, 28097. 26400084 10.1074/jbc.M115.680090 PMC4653669

[13] B. Chu , N. Kon , D. Chen , T. Li , T. Liu , L. Jiang , S. Song , O. Tavana , W. Gu , Nat. Cell Biol. 2019, 21, 579. 30962574 10.1038/s41556-019-0305-6 PMC6624840

[14] B. Do Van , F. Gouel , A. Jonneaux , K. Timmerman , P. Gele , M. Petrault , M. Bastide , C. Laloux , C. Moreau , R. Bordet , D. Devos , J. C. Devedjian , Neurobiol. Dis. 2016, 94, 169. 27189756 10.1016/j.nbd.2016.05.011

[15] B. Yan , Y. Ai , Q. Sun , Y. Ma , Y. Cao , J. Wang , Z. Zhang , X. Wang , Mol. Cell 2021, 81, 355. 33321093 10.1016/j.molcel.2020.11.024

[16] Y. Zhang , J. Shi , X. Liu , L. Feng , Z. Gong , P. Koppula , K. Sirohi , X. Li , Y. Wei , H. Lee , L. Zhuang , G. Chen , Z. D. Xiao , M. C. Hung , J. Chen , P. Huang , W. Li , B. Gan , Nat. Cell Biol. 2018, 20, 1181. 30202049 10.1038/s41556-018-0178-0 PMC6170713

[17] B. Hassannia , P. Vandenabeele , T. Vanden Berghe , Cancer Cell 2019, 35, 830. 31105042 10.1016/j.ccell.2019.04.002

[18] M. J. Hangauer , V. S. Viswanathan , M. J. Ryan , D. Bole , J. K. Eaton , A. Matov , J. Galeas , H. D. Dhruv , M. E. Berens , S. L. Schreiber , F. McCormick , M. T. McManus , Nature 2017, 551, 247. 29088702 10.1038/nature24297 PMC5933935

[19] V. S. Viswanathan , M. J. Ryan , H. D. Dhruv , S. Gill , O. M. Eichhoff , B. Seashore‐Ludlow , S. D. Kaffenberger , J. K. Eaton , K. Shimada , A. J. Aguirre , S. R. Viswanathan , S. Chattopadhyay , P. Tamayo , W. S. Yang , M. G. Rees , S. Chen , Z. V. Boskovic , S. Javaid , C. Huang , X. Wu , Y. Y. Tseng , E. M. Roider , D. Gao , J. M. Cleary , B. M. Wolpin , J. P. Mesirov , D. A. Haber , J. A. Engelman , J. S. Boehm , J. D. Kotz , et al., Nature 2017, 547, 453. 28678785 10.1038/nature23007 PMC5667900

[20] J. Wu , A. M. Minikes , M. Gao , H. Bian , Y. Li , B. R. Stockwell , Z. N. Chen , X. Jiang , Nature 2019, 572, 402. 31341276 10.1038/s41586-019-1426-6 PMC6697195

[21] Y. Zou , W. S. Henry , E. L. Ricq , E. T. Graham , V. V. Phadnis , P. Maretich , S. Paradkar , N. Boehnke , A. A. Deik , F. Reinhardt , J. K. Eaton , B. Ferguson , W. Wang , J. Fairman , H. R. Keys , V. Dancik , C. B. Clish , P. A. Clemons , P. T. Hammond , L. A. Boyer , R. A. Weinberg , S. L. Schreiber , Nature 2020, 585, 603. 32939090 10.1038/s41586-020-2732-8 PMC8051864

[22] Y. Zou , M. J. Palte , A. A. Deik , H. Li , J. K. Eaton , W. Wang , Y. Y. Tseng , R. Deasy , M. Kost‐Alimova , V. Dancik , E. S. Leshchiner , V. S. Viswanathan , S. Signoretti , T. K. Choueiri , J. S. Boehm , B. K. Wagner , J. G. Doench , C. B. Clish , P. A. Clemons , S. L. Schreiber , Nat. Commun. 2019, 10, 1617. 30962421 10.1038/s41467-019-09277-9 PMC6453886

[23] M. D. Maines , Annu. Rev. Pharmacol. Toxicol. 1997, 37, 517. 9131263 10.1146/annurev.pharmtox.37.1.517

[24] X. Fang , H. Wang , D. Han , E. Xie , X. Yang , J. Wei , S. Gu , F. Gao , N. Zhu , X. Yin , Q. Cheng , P. Zhang , W. Dai , J. Chen , F. Yang , H. T. Yang , A. Linkermann , W. Gu , J. Min , F. Wang , Proc. Natl. Acad. Sci. U S A 2019, 116, 2672. 30692261 10.1073/pnas.1821022116 PMC6377499

[25] B. Hassannia , B. Wiernicki , I. Ingold , F. Qu , S. Van Herck , Y. Y. Tyurina , H. Bayir , B. A. Abhari , J. P. F. Angeli , S. M. Choi , E. Meul , K. Heyninck , K. Declerck , C. S. Chirumamilla , M. Lahtela‐Kakkonen , G. Van Camp , D. V. Krysko , P. G. Ekert , S. Fulda , B. G. De Geest , M. Conrad , V. E. Kagan , W. Vanden Berghe , P. Vandenabeele , T. Vanden Berghe , J. Clin. Invest. 2018, 128, 3341. 29939160 10.1172/JCI99032 PMC6063467

[26] Z. Tang , Y. Ju , X. Dai , N. Ni , Y. Liu , D. Zhang , H. Gao , H. Sun , J. Zhang , P. Gu , Redox Biol. 2021, 43, 101971. 33895485 10.1016/j.redox.2021.101971 PMC8099560

[27] S. M. Corsello , R. T. Nagari , R. D. Spangler , J. Rossen , M. Kocak , J. G. Bryan , R. Humeidi , D. Peck , X. Wu , A. A. Tang , V. M. Wang , S. A. Bender , E. Lemire , R. Narayan , P. Montgomery , U. Ben‐David , C. W. Garvie , Y. Chen , M. G. Rees , N. J. Lyons , J. M. McFarland , B. T. Wong , L. Wang , N. Dumont , P. J. O'Hearn , E. Stefan , J. G. Doench , C. N. Harrington , H. Greulich , M. Meyerson , et al., Nat Cancer 2020, 1, 235. 32613204 10.1038/s43018-019-0018-6 PMC7328899

[28] F. Iorio , T. A. Knijnenburg , D. J. Vis , G. R. Bignell , M. P. Menden , M. Schubert , N. Aben , E. Goncalves , S. Barthorpe , H. Lightfoot , T. Cokelaer , P. Greninger , E. van Dyk , H. Chang , H. de Silva , H. Heyn , X. Deng , R. K. Egan , Q. Liu , T. Mironenko , X. Mitropoulos , L. Richardson , J. Wang , T. Zhang , S. Moran , S. Sayols , M. Soleimani , D. Tamborero , N. Lopez‐Bigas , P. Ross‐Macdonald , et al., Cell 2016, 166, 740. 27397505 10.1016/j.cell.2016.06.017 PMC4967469

[29] G. Picco , E. D. Chen , L. G. Alonso , F. M. Behan , E. Goncalves , G. Bignell , A. Matchan , B. Fu , R. Banerjee , E. Anderson , A. Butler , C. H. Benes , U. McDermott , D. Dow , F. Iorio , E. Stronach , F. Yang , K. Yusa , J. Saez‐Rodriguez , M. J. Garnett , Nat. Commun. 2019, 10, 2198. 31097696 10.1038/s41467-019-09940-1 PMC6522557

[30] M. G. Rees , B. Seashore‐Ludlow , J. H. Cheah , D. J. Adams , E. V. Price , S. Gill , S. Javaid , M. E. Coletti , V. L. Jones , N. E. Bodycombe , C. K. Soule , B. Alexander , A. Li , P. Montgomery , J. D. Kotz , C. S. Hon , B. Munoz , T. Liefeld , V. Dancik , D. A. Haber , C. B. Clish , J. A. Bittker , M. Palmer , B. K. Wagner , P. A. Clemons , A. F. Shamji , S. L. Schreiber , Nat. Chem. Biol. 2016, 12, 109. 26656090 10.1038/nchembio.1986 PMC4718762

[31] B. Seashore‐Ludlow , M. G. Rees , J. H. Cheah , M. Cokol , E. V. Price , M. E. Coletti , V. Jones , N. E. Bodycombe , C. K. Soule , J. Gould , B. Alexander , A. Li , P. Montgomery , M. J. Wawer , N. Kuru , J. D. Kotz , C. S. Hon , B. Munoz , T. Liefeld , V. Dancik , J. A. Bittker , M. Palmer , J. E. Bradner , A. F. Shamji , P. A. Clemons , S. L. Schreiber , Cancer Discov. 2015, 5, 1210. 26482930 10.1158/2159-8290.CD-15-0235 PMC4631646

[32] L. Kruidenier , C. W. Chung , Z. Cheng , J. Liddle , K. Che , G. Joberty , M. Bantscheff , C. Bountra , A. Bridges , H. Diallo , D. Eberhard , S. Hutchinson , E. Jones , R. Katso , M. Leveridge , P. K. Mander , J. Mosley , C. Ramirez‐Molina , P. Rowland , C. J. Schofield , R. J. Sheppard , J. E. Smith , C. Swales , R. Tanner , P. Thomas , A. Tumber , G. Drewes , U. Oppermann , D. J. Patel , K. Lee , et al., Nature 2012, 488, 404. 22842901 10.1038/nature11262 PMC4691848

[33] T. Jiang , F. J. Sanchez‐Rivera , Y. M. Soto‐Feliciano , Q. Yang , C. Q. Song , A. Bhuatkar , C. M. Haynes , M. T. Hemann , W. Xue , Hepatology 2021, 74, 233. 33336367 10.1002/hep.31685 PMC8209110

[34] S. L. Freshour , S. Kiwala , K. C. Cotto , A. C. Coffman , J. F. McMichael , J. J. Song , M. Griffith , O. L. Griffith , A. H. Wagner , Nucleic Acids Res. 2021, 49, D1144. 33237278 10.1093/nar/gkaa1084 PMC7778926

[35] W. Li , J. Koster , H. Xu , C. H. Chen , T. Xiao , J. S. Liu , M. Brown , X. S. Liu , Genome Biol. 2015, 16, 281. 26673418 10.1186/s13059-015-0843-6 PMC4699372

[36] B. Wang , M. Wang , W. Zhang , T. Xiao , C. H. Chen , A. Wu , F. Wu , N. Traugh , X. Wang , Z. Li , S. Mei , Y. Cui , S. Shi , J. J. Lipp , M. Hinterndorfer , J. Zuber , M. Brown , W. Li , X. S. Liu , Nat. Protoc. 2019, 14, 756. 30710114 10.1038/s41596-018-0113-7 PMC6862721

[37] A. Ianevski , A. K. Giri , T. Aittokallio , Nucleic Acids Res. 2020, 48, W488. 32246720 10.1093/nar/gkaa216 PMC7319457

[38] A. B. Keenan , S. L. Jenkins , K. M. Jagodnik , S. Koplev , E. He , D. Torre , Z. Wang , A. B. Dohlman , M. C. Silverstein , A. Lachmann , M. V. Kuleshov , A. Ma'ayan , V. Stathias , R. Terryn , D. Cooper , M. Forlin , A. Koleti , D. Vidovic , C. Chung , S. C. Schurer , J. Vasiliauskas , M. Pilarczyk , B. Shamsaei , M. Fazel , Y. Ren , W. Niu , N. A. Clark , S. White , N. Mahi , et al., Cell Syst 2018, 6, 13.29199020

[39] M. B. Sporn , K. T. Liby , Nat. Rev. Cancer 2012, 12, 564. 22810811 10.1038/nrc3278 PMC3836441

[40] J. D. Buenrostro , B. Wu , H. Y. Chang , W. J. Greenleaf , Curr. Protoc. Mol. Biol. 2015, 109, 21291. 10.1002/0471142727.mb2129s109 PMC4374986 25559105

[41] H. S. Kaya‐Okur , S. J. Wu , C. A. Codomo , E. S. Pledger , T. D. Bryson , J. G. Henikoff , K. Ahmad , S. Henikoff , Nat. Commun. 2019, 10, 1930. 31036827 10.1038/s41467-019-09982-5 PMC6488672

[42] A. Panigrahi , B. W. O'Malley , Genome Biol. 2021, 22, 108. 33858480 10.1186/s13059-021-02322-1 PMC8051032

[43] L. Lang , Gastroenterology 2008, 134, 379. 10.1053/j.gastro.2007.12.037 18242200

[44] M. Kudo , R. S. Finn , S. Qin , K. H. Han , K. Ikeda , F. Piscaglia , A. Baron , J. W. Park , G. Han , J. Jassem , J. F. Blanc , A. Vogel , D. Komov , T. R. J. Evans , C. Lopez , C. Dutcus , M. Guo , K. Saito , S. Kraljevic , T. Tamai , M. Ren , A. L. Cheng , Lancet 2018, 391, 1163. 29433850 10.1016/S0140-6736(18)30207-1

[45] R. Rudalska , D. Dauch , T. Longerich , K. McJunkin , T. Wuestefeld , T. W. Kang , A. Hohmeyer , M. Pesic , J. Leibold , A. von Thun , P. Schirmacher , J. Zuber , K. H. Weiss , S. Powers , N. P. Malek , M. Eilers , B. Sipos , S. W. Lowe , R. Geffers , S. Laufer , L. Zender , Nat. Med. 2014, 20, 1138. 25216638 10.1038/nm.3679 PMC4587571

[46] J. Guo , B. Xu , Q. Han , H. Zhou , Y. Xia , C. Gong , X. Dai , Z. Li , G. Wu , Cancer Res. Treat. 2018, 50, 445. 28494534 10.4143/crt.2016.572 PMC5912137

[47] G. Lei , Y. Zhang , P. Koppula , X. Liu , J. Zhang , S. H. Lin , J. A. Ajani , Q. Xiao , Z. Liao , H. Wang , B. Gan , Cell Res. 2020, 30, 146. 31949285 10.1038/s41422-019-0263-3 PMC7015061

[48] A. Pickhaven , Aust. Nurses J. 1975, 4, 4.

[49] X. Sun , Z. Ou , R. Chen , X. Niu , D. Chen , R. Kang , D. Tang , Hepatology 2016, 63, 173. 26403645 10.1002/hep.28251 PMC4688087

[50] R. Wei , Y. Zhao , J. Wang , X. Yang , S. Li , Y. Wang , X. Yang , J. Fei , X. Hao , Y. Zhao , L. Gui , X. Ding , Int. J. Biol. Sci. 2021, 17, 2703. 34345202 10.7150/ijbs.59404 PMC8326123

[51] J. G. Doench , N. Fusi , M. Sullender , M. Hegde , E. W. Vaimberg , K. F. Donovan , I. Smith , Z. Tothova , C. Wilen , R. Orchard , H. W. Virgin , J. Listgarten , D. E. Root , Nat. Biotechnol. 2016, 34, 184. 26780180 10.1038/nbt.3437 PMC4744125

[52] J. Joung , J. M. Engreitz , S. Konermann , O. O. Abudayyeh , V. K. Verdine , F. Aguet , J. S. Gootenberg , N. E. Sanjana , J. B. Wright , C. P. Fulco , Y. Y. Tseng , C. H. Yoon , J. S. Boehm , E. S. Lander , F. Zhang , Nature 2017, 548, 343. 28792927 10.1038/nature23451 PMC5706657

[53] L. Broutier , G. Mastrogiovanni , M. M. Verstegen , H. E. Francies , L. M. Gavarro , C. R. Bradshaw , G. E. Allen , R. Arnes‐Benito , O. Sidorova , M. P. Gaspersz , N. Georgakopoulos , B. K. Koo , S. Dietmann , S. E. Davies , R. K. Praseedom , R. Lieshout , I. J. JNM , S. J. Wigmore , K. Saeb‐Parsy , M. J. Garnett , L. J. van der Laan , M. Huch , Nat. Med. 2017, 23, 1424. 29131160 10.1038/nm.4438 PMC5722201

[54] Z. Xie , A. Bailey , M. V. Kuleshov , D. J. B. Clarke , J. E. Evangelista , S. L. Jenkins , A. Lachmann , M. L. Wojciechowicz , E. Kropiwnicki , K. M. Jagodnik , M. Jeon , A. Ma'ayan , Curr. Protoc. 2021, 1, e90. 33780170 10.1002/cpz1.90 PMC8152575

[55] J. D. Buenrostro , P. G. Giresi , L. C. Zaba , H. Y. Chang , W. J. Greenleaf , Nat. Methods 2013, 10, 1213. 24097267 10.1038/nmeth.2688 PMC3959825

[56] N. E. Sanjana , O. Shalem , F. Zhang , Nat. Methods 2014, 11, 783. 25075903 10.1038/nmeth.3047 PMC4486245

[57] O. Shalem , N. E. Sanjana , E. Hartenian , X. Shi , D. A. Scott , T. Mikkelson , D. Heckl , B. L. Ebert , D. E. Root , J. G. Doench , F. Zhang , Science 2014, 343, 84. 24336571 10.1126/science.1247005 PMC4089965

[58] H. Hagege , P. Klous , C. Braem , E. Splinter , J. Dekker , G. Cathala , W. de Laat , T. Forne , Nat. Protoc. 2007, 2, 1722. 17641637 10.1038/nprot.2007.243

